# Left Insula and Right Middle Temporal Gyrus Dominate Cortical Network Discriminating Arousal‐dependent Emotions

**DOI:** 10.1002/advs.202411790

**Published:** 2025-01-17

**Authors:** Na Clara Pan, Runshi Gao, Kai Ma, Liang Qiao, Duanyu Ni, Tao Yu, Yuping Wang

**Affiliations:** ^1^ Department of Neurology Xuanwu Hospital Capital Medical University Beijing 100053 China; ^2^ Beijing Key Laboratory of Neuromodulation Beijing 100053 China; ^3^ Beijing Institute of Functional Neurosurgery Xuanwu Hospital Capital Medical University Beijing 100053 China; ^4^ Institute of sleep and consciousness disorders Center of Epilepsy Beijing Institute for Brain Disorders Capital Medical University Beijing 100069 China; ^5^ Neuromedical Technology Innovation Center of Hebei Province Shijiazhuang Hebei 050000 China; ^6^ Department of Neurology Hebei Hospital of Xuanwu Hospital Capital Medical University the First Hospital of Hebei Medical University Shijiazhuang Hebei 050000 China

**Keywords:** cognition control, electrophysiology, emotion discrimination, network dynamics, neural basis

## Abstract

Emotion processing is an integral part of everyone's life. The basic neural circuits involved in emotion perception are becoming clear, though the emotion's cognitive processing remains under investigation. Utilizing the stereo‐electroencephalograph with high temporal‐spatial resolution, this study aims to decipher the neural pathway responsible for discriminating low‐arousal and high‐arousal emotions. This study involves 19 patients with pharmacologically resistant epilepsy who participate in a delayed match/mismatch sample task designed to separately assess their ability to discriminate between low‐arousal and high‐arousal emotions. Three groups of 11 brain subregions, with dominant lateralization, compose a network, which is identified as responsible for discriminating arousal‐dependent emotions. The connection of these subregions, leading by the left insula and right middle temporal gyrus, defines the pathways for discriminating emotions with different arousals. Further, the separated network patterns related to emotional discrimination are face‐independent. Overall, the left insula and the right middle temporal gyrus emerge as core components in the network, which plays key roles in the dynamic course for discriminating low‐ and high‐arousal emotions in the human brain.

## Graphical abstract

With the excellent spatial and temporal resolution of SEEG, this work revealed specific subregions responsible for discriminating low‐arousal and high‐arousal emotions. Authors further observed that distinct networks emerged for differentiating these emotions. Additionally, the network for discriminating emotions with different faces is more efficient than that for the same faces.

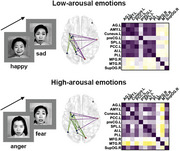

## 1 Introduction

Emotions are psychological states characterized by complex physiological reactions. For centuries, scientists have studied various neural underpinnings of emotions, focusing on three main levels: processing primitive emotional features, emotional learning and the evaluation and integration of multifaceted stimuli, and cognition along with the subjective appraisal of feelings.^[^ ^1^ ^]^ Research in primate and rodent animals has clarified how emotional features are processed in deep subcortical circuits.^[^ ^1^ ^]^ However, less is known about the higher hierarchical‐level procedures, such as emotion‐cognition interactions mediated by neocortical regions.^[^ ^2^ ^]^

Emotional discrimination, apart from recognition, is a cognitive control activity involving goal‐directed processes. It refers to the ability to perceive differences between the emotions of others, as expressed in facial expressions. Deficits in emotional discrimination are well‐documented in disorders such as autism,^[^ ^3^ ^]^ schizophrenia,^[^ ^4^ ^]^ and depression.^[^ ^5^ ^]^ Therefore, uncovering the mechanism underlying emotion discrimination is essential for a comprehensive understanding of emotional functioning in both healthy and disordered states.

An increasing amount of research focuses on the structure and function of the human brain involved in recognizing and discriminating emotional faces,^[^ ^6^ ^]^ often overlooking emotional discrimination. To date, neuroimaging studies have found brain subregions involved in processing emotions. Functional magnetic resonance imaging studies have shown that the limbic system, including the amygdala and hippocampus, as well as the areas of the extended emotional system and visual‐attentional regions are involved in discriminating emotions;^[^ ^7^, ^8^ ^]^ On the other hand, electrophysiological studies have yet revealed dynamics of emotion processing. One event‐related potential study found that angry and happy emotions could elicit stronger responses than neutral emotions;^[^ ^9^ ^]^ while another study using scalp electroencephalography (EEG) did not show any significant differences in discriminating angry, happy, or neutral schematic emotions.^[^ ^10^ ^]^ The conflict results might be due to the low spatial resolution of scalp EEG. The transcranial direct current stimulation of the right and left inferior frontal gyri have been shown to impact the performance of discriminating high (anger) and low (sad) arousal emotions, respectively.^[^ ^11^ ^]^ Zhou et al. used intracranial stereo‐electroencephalography (SEEG) recordings revealing that several cortical regions participate in discriminating facial emotions, including the inferior and middle frontal gyrus, angular gyrus, postcentral gyrus, precuneus, supramarginal gyrus, middle temporal gyrus, and cingulate gyrus.^[^ ^12^ ^]^ Therefore, due to limitations such as the lack of consideration on lateralization, the focus on only happy‐sad emotions, and confounding factors in facial discrimination, the critical issue of how emotions are discriminated in the human brain remains unclear.

Nowadays, emotions are well‐defined and measured using the widely used valence‐arousal space model.^[^ ^13^, ^14^ ^]^ While arousal might not be distinct from valence, most studies have explored the neural basis of emotions across different levels of valence, which range from negative to positive feelings.^[^ ^15^ ^]^ Emotional arousal, defined as a state of physiological activation, has received little attention in previous studies. While emotional arousal is often assessed through self‐report of feelings induced by stimuli,^[^ ^16^ ^]^ various physiological parameters are used to measure it objectively. These include skin conductance^[^ ^17^ ^]^ and amplitudes of event‐related potential (ERP).^[^ ^18^ ^]^ Rather, the functional magnetic resonance imaging data show that emotional arousal is associated with activity in the bilateral middle temporal gyrus, left hippocampus, and left ventrolateral prefrontal cortex.^[^ ^19^ ^]^ However, it is unclear whether arousal activates a specific neural pathway for emotion discrimination.

Since the dominant methods have either high spatial and poor temporal resolution (e.g., functional magnetic resonance imaging) or high temporal and poor spatial resolution (e.g., EEG and magnetoencephalography), the spatio‐temporal mechanism underlying the discrimination of facial emotions is far from fully understood. Here, SEEG with high spatial and temporal resolution was used to investigate the neural basis that contributes to the discrimination of arousal‐dependent emotions. Although SEEG data were obtained from patients with pharmacologically resistant epilepsy, EEG recordings from sites located far away from the ictal zone and without abnormal activity during the interictal period can be considered indicative of normal activity during typical cognition tasks.

Therefore, we simultaneously recorded SEEG signals from the cortical cortex and amygdala in patients with presurgical epilepsy while they participated in tasks to discriminate emotions. To test the emotional discrimination, the delayed‐match/mismatch‐sample paradigm was used, due to the well‐known component of ERPs, N2/N270, which contributes to set a possible time frame for analyzing the procedure of emotional discrimination. This would help us study the neural mechanism of emotion processing more precisely. Human faces expressing both low‐arousal and high‐arousal emotions were used as visual stimuli. Given that there may be diverse pathways for discriminating human faces from facial emotions, we considered both conditions involving the same face and different faces, to understand how the brain discriminates the emotion rather than facial expression.

## 2 Result

**Figure 1:**
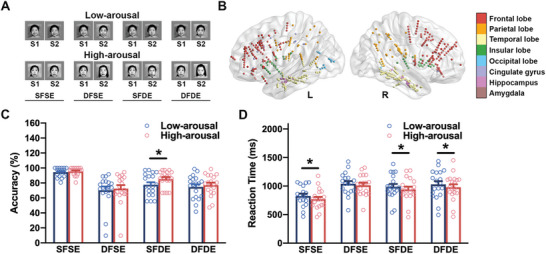
Experiment design and task performance. A) emotional delayed‐matched/mismatched sample tasks. Example conditions of paired S1 and S2 stimuli contained low‐arousal emotions (upper panel) and high‐arousal emotions (bottom panel), respectively. Regardless to the emotional arousal, for condition SFSE, the face of S1 was the same with the face of S2, and the emotion of S1 was the same with S2; for DFSE, the face of S1 was different from the face of S2, and the emotion of S1 was the same with S2; for SFDE, the face of S1 was the same with the face of S2, and the emotion of S1 was different from S2; for condition DFDE, the face of S1 was different from the face of S2, and the emotion of S1 was also different from S2. B) schematics of 586 recording sites located in the left and right hemispheres. Sites were colored by regional groups, including the frontal lobe (red), parietal lobe (orange), temporal lobe (light yellow), insular lobe (green), occipital lobe (sky blue), cingulate lobe (medium purple), the hippocampus regions (violet), and the amygdala (brown). C) the accuracy of participants executing tasks, including four conditions of low‐arousal (blue) and high‐arousal (red) emotion, respectively. The circle indicated the mean accuracy of individual participant in performing tasks in each condition. Data were presented as mean ± SEM after a paired t‐test. For SFSE, *p* = 0.5226, *t* = 0.6520; for DFSE, *p* = 0.6971, *t* = 0.3959; for SFDE, ^*^ *p* = 0.0131, *t* = 2.753; for DFDE, *p* = 0.2785, *t* = 1.117. D) the reaction time of participants executing tasks, including four conditions of low‐arousal (blue) and high‐arousal (red) emotion, respectively. The circle indicated the mean reaction time of individual participant in performing tasks in each condition. Data were presented as mean ± SEM after a paired *t*‐test. For SFSE, ^*^ *p* = 0.0113, *t* = 2.821; for DFSE, *p* = 0.4918, *t* = 0.7027; for SFDE, ^*^ *p* = 0.0444, *t* = 2.161; for DFDE, ^*^ *p* = 0.0428, *t* = 2.180.

Nineteen patients with pharmacologically resistant epilepsy underwent SEEG electrode implantation for seizure focus mapping. These patients were assessed to have normal cognitive abilities after clinical evaluation. The patients participated in a delayed matched/mismatched sample task, which featured low‐arousal or high‐arousal emotional faces as sequential stimuli, that is the first stimulus as S1 and the second stimulus as S2, in four conditions: S1 showed the same face and same emotion as S2 (“SFSE”), S1 showed the same face as S2 but different emotions from S2 (“SFDE”), S1 showed different faces from S2 but same emotion as S2 (“DFSE”), and S1 showed different faces and different emotions from S2 (“DFDE”) (**Figure** **1**A). The local field potentials from 586 contacts distributed in 26 subregions of the left and right hemispheres (Figure 1B; Table S1, Supporting Information) were recorded.

### 2.1 Arousal‐Dependent Faces Discriminate Behavioral Reactions in Tasks

Given the impact of emotional arousal on cognitive activity, we first examined the behavioral responses, including the accuracy and reaction time of individual participants, while they judged the incongruency of low‐ and high‐arousal faces, respectively. The accuracy across the four conditions revealed that participants had higher scores when discriminating incongruencies in high‐arousal faces than in low‐arousal faces (Figure 1C). Specifically, for the SFDE condition, the accuracy for processing incongruencies in low‐arousal faces was 77.63 ± 3.36%, while for high‐arousal faces it was 85.38 ± 2.51% (*p* = 0.0131, *n* = 19). However, no significant difference in accuracy between low‐arousal and high‐arousal faces was observed in the other three conditions (SFSE, DFSE, and DFDE).

For reaction time, the participants demonstrated shorter reaction times when addressing incongruencies in high‐arousal faces than in low‐arousal faces in the SFSE, SFDE, and DFDE conditions (Figure 1D). For condition SFSE, participants took 827.6 ± 40.06 ms to judge the congruency of low‐arousal faces, versus 770.6 ± 40.37 ms of high‐arousal (*p* = 0.0113, *n* = 19). For condition SFDE, 987.7 ± 49.73 ms were spent to judge the congruency of low‐arousal faces, versus 941.5 ± 47.53 ms of high‐arousal (*p* = 0.0444, *n* = 19). For condition DFDE, processing the congruency of low‐arousal faces cost 1029 ± 57.54 ms, versus 979.4 ± 53.05 ms of high‐arousal (*p* = 0.0428, *n* = 19).

Thus, participants had shorter reaction times and higher accuracies when processing high‐arousal emotional incongruencies compared to low‐arousal emotions. The significant differences in the participants' behavioral reactions when processing incongruencies in low‐arousal versus high‐arousal faces suggested that arousal‐dependent emotional faces induced different cognitive activities in the brain, which might be associated with distinct neural mechanisms.

### 2.2 Distinct Brain Subregions Involved in Arousal‐Related Emotional Discrimination

To characterize the underlying neural activities of the incongruency processing induced by arousal‐dependent faces, intracranial ERPs in the brain were examined. The negative component of event‐related potential, named N2/N270, is markable and widely used for evaluating incongruent visual stimuli.^[^ ^20^ ^]^ Therefore, the potential during S2 is a key focus for studying emotional discrimination.

**Figure 2:**
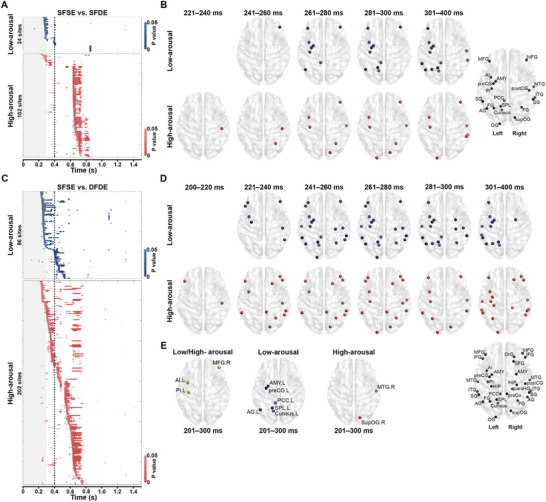
Chronological distributions in processing emotional incongruencies. A) heatmaps of *p* values from the permutation test of the SEEG amplitudes between the condition SFSE and SFDE in processing low‐arousal (blue, upper panel) and high‐arousal (red, bottom panel), respectively. Each row represented one recording site. The values of *p *< 0.05 were implicated as dark blue and red, respectively. The value of *p* > 0.05 were shown as the white background in the heatmap. The vertical gray rectangle indicated the durations of S2 (300 ms). The dotted line indicated the timepoint of 400 ms from S2 initiation. The recording sites were ordered as the occurrence time of the FDR‐corrected *p* value < 0.05 lasting for at least 20 ms (*n* = 34 sites in low‐arousal emotion and *n* = 102 sites in high‐arousal emotion). B) distributions of recording sites participating in processing low‐arousal (blue, upper panel) and high‐arousal (red, bottom panel) emotional incongruencies in the continuous timeframe, according to the *p* value shown in (A). The site label was shown in the template of left (.L) and right (.R) hemispheres. C) heatmaps of *p* values from the permutation test of the SEEG amplitudes between the condition SFSE and DFDE in processing low‐arousal (blue, upper panel) and high‐arousal (red, bottom panel), respectively. Each row represented one recording site. The value of *p* < 0.05 were implicated as dark blue and red, respectively. The value of *p* > 0.05 was shown as the white background in the heatmap. The vertical gray rectangle indicated the durations of S2 (300 ms). The dotted line indicated the timepoint of 400 ms from S2 initiation. The recording sites were ordered as the occurrence time of the FDR‐corrected *p* value < 0.05 lasting for at least 20 ms (*n* = 86 sites in low‐arousal emotion and n = 202 sites in high‐arousal emotion). D) distributions of recording sites participating in processing low‐arousal (blue, upper panel) and high‐arousal (red, bottom panel) emotional incongruencies in the continuous timeframe, according to the *p* value shown in (C). The site label was shown in the template of the left (.L) and right (R) hemispheres. E) active regions responsible for various conditions after S2 onset. Left panel, distributions of recording sites (yellow) participating in processing both low‐arousal and high‐arousal emotional incongruencies in condition SFDE (vs SFSE) and DFDE (vs SFSE) during 201–300 ms from S2 initiation. Middle panel, distributions of sites (blue) participating in processing only low‐arousal emotional incongruencies in condition SFDE (vs SFSE) and DFDE (vs SFSE). Right panel, distributions of sites participating in processing only high‐arousal emotional incongruencies in condition SFDE (vs SFSE) and DFDE (vs SFSE). Abbreviation: AG, angular gyrus; AI, anterior insula; AMY, amygdala; FG, fusiform gyrus; HIP, hippocampus; IFG, inferior frontal gyrus; ITG, inferior temporal gyrus; MFG, middle frontal gyrus; MTG, middle temporal gyrus; paraHG, parahippocapus gyrus; PCC, posterior cingulate cortex; PI, posterior insula; postCG, postcentral gyrus; preCG, precentral gyrus; preCu, precuneus; SFG, superior frontal gyrus; SG, supramarginal gyrus; SPL, superior parietal lobule; supOG, superior occipital gyrus; OG, occipital gurys; OrG, orbital gyrus.

In both the low‐arousal and high‐arousal groups and across the four conditions (SFSE, DFSE, DFDE, and DFDE), ERPs of each site were extracted from correct‐response trials and grouped by subregions covering these sites (Table S1, Supporting Information). To detect differences related to emotional incongruency, two scenarios were considered: incongruent emotions from the same face (condition SFDE) and incongruent emotions from different faces (condition DFDE). By comparing the ERPs of SFDE or DFDE with the baseline of condition SFSE (same emotion from the same face) within each subregion, a permutation test identified sites that showed *p* < 0.05 for lasting > 20 ms after S2 onset, in both of the low‐arousal and high‐arousal groups (**Figure** **2**). These sites were considered as candidates for incongruency processing in two situations.

Of the 1029 sites comparing the condition SFSE with SFDE, 34 sites showed significant differences in the low‐arousal group after S2 onset (upper panel in Figure 2A). Among these, 28 sites exhibited differences within the first 400 ms following S2 onset. These 28 sites were distributed across 14 subregions, primarily in the left hemisphere (upper panel in Figure 2B), in a chronological pattern. Typically, the right middle frontal gyrus was the first region to react in incongruency processing during the 241–260 ms after S2 onset. In contrast, in the high‐arousal group, 102 sites showed significant differences after S2 onset (bottom panel in Figure 2A). Among these, only 12 sites distributed across ten subregions, mainly in the right hemisphere, demonstrated differences within the first 400 ms following S2 onset (bottom panel in Figure 2B). Notably, earlier than in the low‐arousal group, the right postcentral gyrus was the first region involved in processing incongruency, starting at 221–240 ms after S2 onset.

Additionally, comparing the condition SFSE with DFDE, which is more complicated than SFSE versus SFDE, revealed more sites involved in incongruency processing in the brain. Of the 1029 recorded sites, 86 showed significant differences in the low‐arousal group after S2 onset (upper panel in Figure 2C). Among these, 63 sites exhibited differences within the first 400 ms following S2 onset. These sites were distributed across twenty‐two subregions, primarily in the left hemisphere (upper panel in Figure 2D), in a chronological manner. Typically, several regions, including the right middle frontal gyrus, were the first to react to incongruency processing during the 221–240 ms after S2 onset. In contrast, in the high‐arousal group, 202 sites showed significant differences after S2 onset (bottom panel in Figure 2C). Among them, 86 sites distributed across twenty‐four subregions, mainly in the right hemisphere, showed differences in the first 400 ms following S2 onset (bottom panel in Figure 2D). Notably, brain activity related to processing emotional incongruency began earlier in the high‐arousal group than in the low‐arousal group. Specifically, the right postcentral gyrus and left inferior frontal gyrus were the first brain regions to show this activity, starting between 200–220 ms after S2 onset.

In summary, distinct brain subregions are involved in arousal‐related emotional discrimination. More sub‐regions participate in emotional discrimination when the faces are different than when they are the same. Both in the SFSE versus SFDE, and SFSE versus DFDE comparisons, regions responded earlier to high‐arousal stimuli than to low‐arousal stimuli. The regions in the left hemisphere predominantly participated in low‐arousal emotional discrimination, whereas the right hemisphere mainly participated in high‐arousal emotional discrimination.

Notably, a pattern was observed in regions involved in cognitive activities (Figure 2E). The left anterior insular, left posterior insular, and right middle frontal gyri seemed to be consistently engaged in processing emotional incongruency, regardless of the situation or emotional arousal. This indicated that these three regions were fundamental neural basis for emotional discrimination. Moreover, six regions clustering in the left hemisphere, including the left amygdala, precentral gyrus, posterior cingulate cortex, superior parietal lobule, angular gyrus, and cuneus, were involved only in processing low‐arousal emotional incongruency, whatever in the condition SFDE or DFDE. Accordingly, two regions within the right hemisphere, the right middle temporal gyrus, and the superior occipital gyrus, were involved only in processing high‐arousal emotional incongruency. The representative ERPs of each region in each situation are shown in Figure S1 (Supporting Information). Thus, the results suggested that the eleven core regions (we called them “nodes” in the following) mentioned above played a critical role in arousal‐dependent emotional discrimination.

### 2.3 Distinct Connections Separate the Arousal‐Dependent Emotional Incongruency Processing

To explore the neural mechanisms underlying arousal‐dependent emotional discrimination, the neural basis of the local networks consisting of the 11 critical nodes mentioned above was unveiled. We checked the connectivity between the nodes during this time.

**Figure 3:**
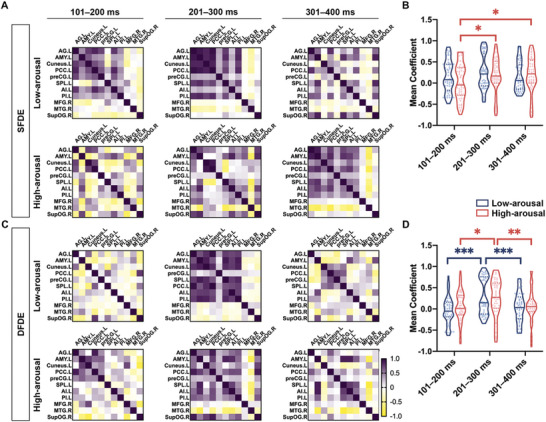
Correlation of core subregions in the low‐arousal and high‐arousal emotional incongruencies processing. A,C) Heatmaps of pairwise correlation of SEEG between two subregions during 101–200 ms, 201–300 ms, and 301–400 ms from S2 onset, in condition SFDE **A**) and DFDE **C**), with low‐arousal (upper in each panel) and high‐arousal (bottom in each panel). Spearman correlation was applied. The correlation coefficient with *p* < 0.05 was considered an effective connection. The correlation coefficient with *p* > 0.05 was considered as ineffective connection, as the coefficient showed as value 0. The averaged coefficient was shown in the heatmap, as the dark purple indicated the positive correlation and the yellow indicated the negative correlation. B,D) statistics of averaged coefficient of connections within core nodes in condition SFDE B) and DFDE D), which was indicated in (A) and (B). A mixed‐effects model was applied to analyze the effect of “time” and “arousal” on the integral connections between low‐arousal and high‐arousal emotional incongruency processing. In condition SFDE (**C**), *p* = 0.1638 for interaction between the effect of “time” and “arousal” (*F* (2, 216) = 1.824); *P = 0.0227 for the effect of “time” (*F* (1.796, 194) = 4.045); and *p* = 0.3141 for the effect of “arousal” (*F* (1, 108) = 1.023). The posthoc test was applied in “low‐arousal” versus “high‐arousal,” that is, *p* = 0.0699 in 101–200 ms; *p* = 0.4288 in 201–300 ms; and *p* = 0.6720 in 301–400 ms. The posthoc multiple comparison test was applied in low‐arousal, *p* = 0.1059 (101–200 ms vs 201–300 ms), *p* = 0.9937 (101–200 ms vs 301–400 ms), and *p* = 0.0877 (201–300 ms vs 301–400 ms); in high‐arousal (red star), ^*^ *p* = 0.0136 (101–200 ms vs 201–300 ms), ^*^ *p* = 0.0365 (101–200 ms vs 301–400 ms), and *p* = 0.9107 (201–300 ms vs 301–400 ms). In condition DFDE D), *p* = 0.3893 for interaction between the effect of “time” and “arousal” (*F* (2, 210) = 0.9477); ^***^ *p* < 0.0001 for the effect of “time” (*F* (1.962, 206.0) = 16.59); and *p* = 0.4075 for the effect of “arousal” (*F* (1, 108) = 0.6916). The posthoc test was applied in “low‐arousal” versus “high‐arousal” (black star), that is, *p* = 0.0984 in 101–200 ms; *p* = 0.7743 in 201–300 ms; and *p* = 0.6014 in 301–400 ms. The posthoc multiple comparison test was applied in low‐arousal (blue star), ^***^ *p* < 0.0001 (101–200 ms vs 201–300 ms), *p* = 0.2818 (101–200 ms vs 301–400 ms), and ^***^ *p* = 0.0006 (201–300 ms vs 301–400 ms); in high‐arousal (red star), *p* = 0.0128 (101–200 ms vs 201–300 ms), *p* = 0.9986 (101–200 ms vs 301–400 ms), and ^**^ *p* = 0.0026 (201–300 ms vs 301–400 ms). Abbreviation: AG, angular gyrus; AI, anterior insula; AMY, amygdala; MFG, middle frontal gyrus; MTG, middle temporal gyrus; PCC, posterior cingulate cortex; PI, posterior insula; preCG, precentral gyrus; SG, supramarginal gyrus; SPL, superior parietal lobule; supOG, superior occipital gyrus.

Here, the activities of condition SFDE were featured by subtracting the corresponding averaged ERP of SFSE from that of SFDF for each site from the eleven nodes, including the angular gyrus, amygdala, cuneus, posterior cingulate cortex, precentral gyrus, superior parietal lobule, and anterior and posterior insular in the left hemisphere, and the middle frontal gyrus, middle temporal gyrus, and superior occipital gyrus in the right hemisphere. Next, Spearman's correlation was used to calculate the connectivity between sites. Thus, in the SFDE condition, the connectivity between the nodes was determined by averaging the correlation coefficients between sites within each node (**Figure** **3**A). In low‐arousal emotional discrimination, the correlation within the left hemisphere was more positive between 101 and 300 ms, with a mixed correlation observed afterward. In contrast, in high‐arousal emotional discrimination, a mixed correlation persisted between 101 and 300 ms, with positive connections emerging in the left hemisphere after 300 ms. The integrated connection strength of the 11‐node network showed no significant difference between the low‐arousal and high‐arousal emotional discrimination in each timeframe during S2. However, the integrated connection strength increased 200 ms after S2 onset, when the incongruent high‐arousal emotions were processed (Figure 3B).

In the DFDE condition, in both low‐ and high‐arousal emotional discrimination, a dominant positive correlation was observed from 201 to 300 ms (Figure 3C). The integrated connection strength also showed no significant difference between low‐ and high‐arousal emotional discrimination in each timeframe during S2; however, in both low‐ and high‐arousal emotional discrimination, the integrated connection strength increased 200 ms after S2 onset (Figure 3D). Therefore, the connection of the 11‐node network might modulate different pathways for processing incongruencies induced by low‐arousal and high‐arousal in the human brain.

**Figure 4:**
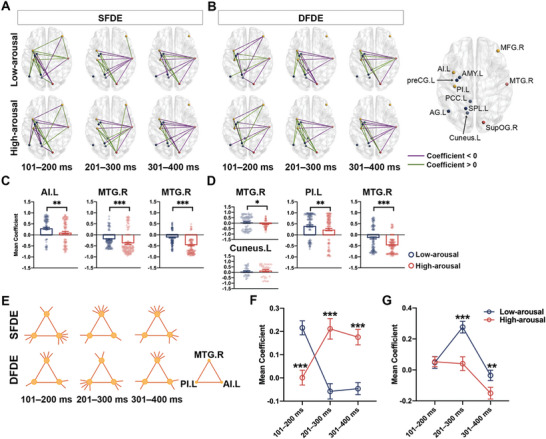
Discrepant connectivity within core subregions participating in processing emotion incongruencies. A,B) connection correlation within three groups of subregions along S2 appearance, in SFDE A) and DFDE condition B), respectively. The schematics of connection showed significantly different connectivity between the low‐arousal and high‐arousal emotional stimuli, after the Mann–Whitney test with *p* < 0.05. The positive correlation was indicated as a green line and the negative correlation was indicated as a purple line. Three groups of nodes were shown, including yellow nodes participating in processing both low‐arousal and high‐arousal emotional incongruencies; blue nodes participating in processing only low‐arousal emotional incongruencies; and red nodes participating in processing only high‐arousal emotional incongruencies. The node label was shown in the template of left (.L) and right (.R) hemisphere. C,D) statistics of averaged coefficient of connections of nodes within the highest degree centrality, in SFDE (C) and DFDE (D) condition, which was indicated in (A‐B). Mann–Whitney test was applied to analyze the integral connections of a core node with other nodes, between low‐arousal and high‐arousal emotional incongruency processing. ^*^ *p* < 0.05, ^**^ *p* < 0.01, and ^***^ *p* < 0.001. E) discrepant connectivity pattern involving centrality nodes, including MTG.R, PI.L, and AI. The circles dots indicated the core nodes, long lines indicated the direct connection between two nodes, and short lines indicated the links between the centrality node and other nodes. The node label was shown in the template of left (.L) and right (.R) hemispheres. F,G) averaged coefficient of connections of networks in A, in SFDE (F) and DFDE (G) condition. Mann‐Whitney test was applied to analyze the integral connections between low‐arousal and high‐arousal emotional incongruency processing. ^**^ *p* < 0.01, and ^***^ *p* < 0.001. Abbreviation: AG, angular gyrus; AI, anterior insula; AMY, amygdala; MFG, middle frontal gyrus; MTG, middle temporal gyrus; PCC, posterior cingulate cortex; PI, posterior insula; preCG, precentral gyrus; SG, supramarginal gyrus; SPL, superior parietal lobule; supOG, superior occipital gyrus.

By chronologically comparing the connection between each pair of nodes in the low‐arousal case with the high‐arousal case, significant differences were observed under the SFDE (**Figure** **4**A) and DFDE (Figure 4B) conditions. These connections, due to either opposite connection directions or distinct connection strengths, differentiated the incongruency processing of low‐arousal emotions from that of high‐arousal emotions (Figure S2, Supporting Information).

When discriminating emotions from the same face (SFDE), during 101–200 ms, the left anterior insula exhibited the highest degree centrality, having the most connections with other nodes (degree = 7). The strength of these connections in the left anterior insula was greater for low‐arousal emotions than for high‐arousal emotions. After 200 ms of S2 onset, the right middle temporal gyrus exhibited the highest degree centrality (degree = 8) and had weaker negative connections when processing low‐arousal emotions than high‐arousal emotions (Figure 4C). However, in the situation of discriminating emotions with different faces (DFDE), during the early 101–200 ms, both the right middle temporal gyrus and left cuneus had the most links with other nodes (degree = 6). However, only the average coefficient of the right middle temporal gyrus showed significant differences between low‐ and high‐arousal emotion discrimination. After 200 ms of the S2 onset, the left posterior insula exhibited the highest degree centrality (degree = 7), showing a higher strength of positive connections in the low‐arousal case than in the high‐arousal case. In the late 301–400 ms, the right middle temporal gyrus acted as the critical node (degree = 7), with a lower strength of negative connections in the low‐arousal case than in the high‐arousal case (Figure 4D), which was consistent with that in the SFDE.

These results suggested that the anterior and posterior insula in the left hemisphere and the middle temporal gyrus in the right hemisphere played central roles in discriminating arousal‐dependent emotions. Three nodes were identified as core nodes involved in arousal‐dependent emotional discrimination, both under conditions of the same face (SFDE) and different faces (DFDE) (Figure 4E). In the SFDE condition, the integrated connections with the triple nodes showed a higher strength of high‐arousal emotional processing than that of low‐arousal from 200 ms of S2 onset (Figure 4F). In contrast, in the DFDE condition, the integrated connection with triple nides demonstrated a lower strength of high‐arousal emotional processing than that of low‐arousal during the same timeframe (Figure 4G).

Thus, the discrepant connection direction and strength within the 11‐node network distinguished the processing of incongruencies between low‐arousal and high‐arousal emotions via respective pathways, which were centralized by the left insula and right middle temporal gyrus within the network. Both the left anterior and posterior insula exhibited positive connections with other nodes, whereas the right middle temporal gyrus exhibited negative connections. It indicated that the left insula and the right middle temporal gyrus would act as an excitatory and inhibitory function within the network, respectively. The brain employs stronger positive and weaker negative connections to discriminate the low‐arousal emotions compared with the high‐arousal emotions.

### 2.4 Network Pattern for Processing Emotion‐Relevant and Emotion‐Irrelevant Incongruencies

**Figure 5:**
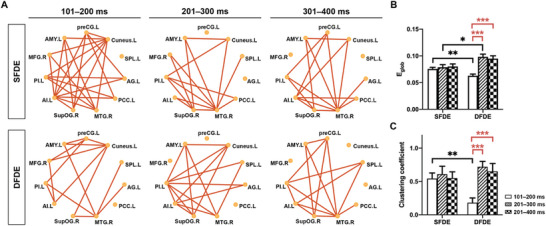
Network evolution of SEEG connections in processing emotion incongruencies. A) connectivity pattern of networks consisting of core nodes, which had shown significant different correlating coefficients between the low‐arousal and high‐arousal emotional incongruency processing, in condition SFDE (upper panel) and DFDE (bottom panel), respectively. The circles dots indicated the core nodes and lines indicated the direct connection between two nodes. B) global efficiency (E_glob_) of each pattern of networks in condition SFDE and DFDE in various of time durations. In each condition, one‐way ANOVA was applied to analyze the time effect on the E_glob_, For SFDE, *p* = 0.7575 and F (DFn, Dfd) = 0.2809 (2,15). The posthoc multiple comparisons showed *p* = 0.5976 (101–200 ms vs 201–300 ms), *p* = 0.4331 (101–200 ms vs 301–400 ms), and *p* = 0.8004 (201–300 ms vs 301–400 ms). For DFDE, ^***^ *p* < 0.0001 and F (DFn, Dfd) = 0.1619 (2,23). The posthoc multiple comparisons (red stars) showed ^***^ *p* < 0.0001 (101–200 ms vs 201–300 ms), ^***^ *p* < 0.0001 (101–200 ms vs 301–400 ms), and *p* = 0.6116 (201–300 ms vs 301–400 ms). Between conditions (SFDE vs DFDE, black stars), an unpaired *t*‐test was used. In 101–200 ms, ^**^ *p* = 0.0085, t = 2.951, df = 18; in 201–300 ms, ^*^ *p* = 0.0145, t = 2.762, df = 15; and in 301–400 ms, *p* = 0.0551, t = 2.080, df = 15. C) clustering coefficient of each pattern of networks in condition SFDE and DFDE in various time durations. In each condition, one‐way ANOVA was applied to analyze the time effect on the clustering coefficient, For SFDE, *p* = 0.8749 and F (DFn, Dfd) = 0.1344 (2,25). The posthoc multiple comparisons showed *p* = 0.6345 (101–200 ms vs 201–300 ms), *p* = 0.9540 (101–200 ms vs 301–400 ms), and *p* = 0.6836 (201–300 ms vs 301–400 ms). For DFDE, ^***^ *p* = 0.0004 and F (DFn, Dfd) = 11.45 (2,23). The posthoc multiple comparisons (red stars) showed ^***^ *p* = 0.0003 (101–200 ms vs 201–300 ms), ^***^ *p* = 0.0010 (101–200 ms vs 301–400 ms), and *p* = 0.6089 (201–300 ms vs 301–400 ms). Between conditions (SFDE vs DFDE, black stars), an unpaired *t*‐test was used. In 101–200 ms, ^**^ *p* = 0.0043, t = 3.264, df = 18; in 201–300 ms, *p* = 0.4643, t = 0.7509, df = 15; and in 301–400 ms, *p* = 0.5058, t = 0.6818, df = 15.

When discriminating emotion using the same face (SFDE), the emotion was the task‐relevant feature. In contrast, when different faces were used (DFDE), the faces were the task‐irrelevant feature, though they still contributed to incongruency processing. Our study identified distinct 11‐node network patterns for the low‐ and high‐arousal emotional discrimination in both SFDE and DFDE conditions (**Figure** **5**A).

At each timeframe, the SFDE and DFDE networks exhibited typical graphic patterns with links between each two nodes. The global efficiency, which was used to describe the efficiency of information transmission in the network, indicated a lower transmission ability of DFDE than that of SFDE in the early time and then a higher transmission ability of DFDE than that of SFDE 201–300 ms after S2 onset. The transmission efficiency was sustained over time in the SFDE condition; however, it increased by 200 ms after S2 onset in the DFDE condition (Figure 5B).

The clustering coefficient, which indicates the network grouping, was lower for DFDE than for SFDE. Clustering was sustained over time in the SFDE condition; however, clustering increased by 200 ms after S2 onset in the DFDE condition (Figure 5C).

Combining the global efficiency and clustering coefficient between the SFDE and DFDE conditions during 201–300 ms after the S2 onset, the network discriminating arousal‐dependent emotions with different faces was found to be more efficient than that with the same face. The chronological efficiency of the network in the SFSE condition was more stable. In contrast, the network's efficiency for discriminating faces in the DFDE condition was both earlier and more variable than its efficiency for discriminating emotions.

**Figure 6:**
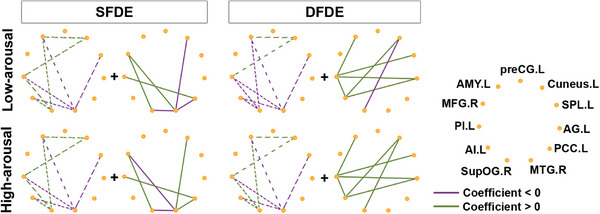
Network separation of SEEG connection in processing arousal‐dependent emotion during 201–300 ms. Connectivity networks, which were consisting of core nodes showed significantly different correlating coefficients between the low‐arousal (upper panel) and high‐arousal (bottom panel) emotional incongruency processing, in condition, SFDE (left panel) and DFDE (right panel), respectively, were separated by the fixed connection pattern (dashed line) and alterable connection pattern (solid line). The circles dots indicated the core nodes and lines indicated the direct connection between two nodes. The positive correlation was indicated as a green line and the negative correlation was indicated as a purple line. The site label was shown in the template of left (.L) and right (.R) hemisphere. Abbreviation: AG, angular gyrus; AI, anterior insula; AMY, amygdala; MFG, middle frontal gyrus; MTG, middle temporal gyrus; PCC, posterior cingulate cortex; PI, posterior insula; preCG, precentral gyrus; SG, supramarginal gyrus; SPL, superior parietal lobule; supOG, superior occipital gyrus.

Notably, the network functions during 201–300 ms could be divided into fixed and alterable connection patterns (**Figure** **6**). The fixed connection pattern, including those connections clustering in the left anterior and posterior insula, right supraoccipital gyrus, middle temporal gyrus, left amygdala, cuneus, and superior parietal lobules, served as the basal connection pattern of processing emotions irrespective of the facial condition and arousal levels. In the SFDE condition, the fixed connection pattern showed that the differences in processing high‐arousal and low‐arousal emotions mainly lay in changes in connection strength. In the DFDE condition, the fixed connection pattern showed oppositely directed connections with the right supraoccipital gyrus between low‐ and high‐arousal emotions, and the remained differences were also changes in connection strength. Thus, the distinctly different connection direction of the right supraoccipital gyrus with other nodes might have been the reason for incongruent faces.

On the other hand, the alterable connections might serve as regulatory connections. In the condition SFDE, the alterable connections included those clustered in the right middle frontal gyrus, supraoccipital gyrus, middle temporal gyrus, left posterior insula, cuneus, and posterior cingulate cortex, determined the discrimination of emotional arousals. However, the alterable connection patterns in the condition DFDE, including those clustered in the left insula, amygdala, cuneus, superior parietal lobules, angular gyrus, and right supraoccipital gyrus, might be involved in tuning the processing of incongruencies in both arousal‐dependent and face‐relevant emotions.

Thus, the results indicated that when discriminating double features (face and emotion), both the fixed and alterable connections involving the right supraoccipital gyrus showed opposite patterns for low‐arousal and high‐arousal emotions. In contrast, when discriminating single features (emotion), only the alterable connections with the right middle temporal gyrus exhibited opposite patterns for low‐arousal and high‐arousal emotions.

## 3 Discussion

Our results provide crucial electrophysiological evidence for understanding how incongruent emotions with low and high arousal levels are processed differently. A patterned distribution of subregions was observed: the left hemisphere was predominantly involved in processing the incongruencies in low‐arousal emotions, while most sites in the right hemisphere played a major role in processing high‐arousal emotional discrimination. Within these subregions, an 11‐node network was identified, including the left anterior insula, left posterior insula, right middle frontal gyrus, left amygdala, precentral gyrus, posterior cingulate cortex, superior parietal lobule, angular gyrus, cuneus, right middle temporal gyrus, and superior occipital gyrus. This network demonstrated distinct pathways for the discrimination of arousal‐dependent emotions with markable connection properties. Furthermore, a positive connection with the left insula and a negative connection with the middle temporal gyrus differentiated low‐arousal from high‐arousal emotional discrimination. Additionally, our results characterized an 11‐node network for discriminating arousal‐dependent emotions, whether the conditions involved the same or different human faces.

This study aimed to determine whether distinct brain responses could be observed when processing incongruencies in low‐arousal versus high‐arousal emotions. Focusing on the significant component of incongruence, N2/N270, we examined the dynamic processes involved in discriminating low‐arousal and high‐arousal emotions under the SFDE and DFDE conditions, particularly during the 201–300 ms interval after S2 onset. Although some subregions overlapped between arousal or conditions, the three groups of subregions played distinct roles. The left insula and right middle frontal gyrus served as foundational nodes, regardless of the arousal level and emotional face type.

According to the James–Lange theory of emotion, humans differentiate emotional states by generating interoceptive responses that reflect changes in autonomic nervous system activity, which has historically been associated with the limbic system, including the insula, especially the anterior insula.^[^ ^21^ ^]^ Apart from the anterior‐posterior distinctions, the insular cortex can also be divided dorsal‐ventrally. The agranular regions have been proven to contribute to higher‐order cognitive and emotion processing.^[^ ^22^ ^]^ The posterior insula, it exhibits the strongest afferent and efferent connectivity with the amygdala, which plays a crucial role in emotional processing and regulation.^[^ ^23^ ^]^ In addition to its role in emotion processing, the insula, including the anterior and posterior parts, is also a critical region for processing incongruent information.^[^ ^12^, ^24^ ^]^ This is consistent with the sensitivity of the insula to changes in salience during cognitive activity.^[^ ^25^ ^]^ The insula is also involved in social decision‐making.^[^ ^26^ ^]^ Hence, combined with our evidence, the left insula stands at the crossroads of sensation and emotion, integrating these streams of information to guide decisions.^[^ ^27^ ^]^

Similarly, the right middle frontal gyrus, which covers most of the dorsolateral prefrontal and anterior ventrolateral prefrontal cortices, has rich connections with subregions involved in emotion processing, such as the insula, amygdala, and hypothalamus, as well as those responsible for cognition control, such as the hippocampus, cingulate cortex, and medial prefrontal cortex.^[^ ^28^ ^]^ The lateral prefrontal lobe is thought to play a critical role in regulating emotional responses, goal‐directed behavior, and even emotion‐cognition interactions.^[^ ^28^, ^29^ ^]^ Therefore, as the core node of the frontoparietal network,^[^ ^30^ ^]^ the right middle frontal gyrus possibly serves as a key controller in the top‐down processing of emotions, cooperating with the insula.

The middle‐to‐posterior lobes mainly participated in discriminating arousal‐dependent emotions, with dominant lateralization of the left or right hemispheres for low‐arousal or high‐arousal, respectively. Furthermore, it seems that higher arousal emotions require fewer subregions to process discrimination. Considering the network connections, those involving the right middle temporal and supraoccipital gyrus are likely to play a more prominent role than connections with the seven left‐sided nodes, which was specific to low‐arousal emotional discrimination. The separating network shown in Figure 6 further supports the importance of the two right subregions in feature‐related arousal‐dependent discrimination. Specifically, the right middle temporal gyrus, with the highest degree of centrality within networks, can be considered the core node for processing incongruent emotions between low‐ and high‐arousal states. Evidence from studies on lesions and disorders involving temporal abnormalities has shown that the middle temporal gyrus acts as a central brain region for the cognitive processing of emotions.^[^ ^31^, ^32^ ^]^ Leveraging the high temporal and spatial resolution of SEEG, our results supported the notion that the right middle temporal gyrus directly participated in high‐arousal emotional discrimination, and its distinct network connections are responsible for the differential processing of low‐ and high‐arousal emotions.

Considering the functional connection between the insula and the middle temporal gyrus,^[^ ^33^ ^]^ and combining the cognitive functions of the insula, middle frontal gyrus, and middle temporal gyrus in emotional processing, it could be inferred that the brain's network—comprising the insula, which acts as an active interface for integrating sensory, emotional and incongruent information;^[^ ^27^ ^]^ the middle temporal gyrus, which refers to emotional regulation^[^ ^34^ ^]^ and memory;^[^ ^35^ ^]^ and the lateral prefrontal cortex, which supports the top‐down modulation of task‐relevant processes^[^ ^36^ ^]^—likely represents the primary pathway for processing arousal‐dependent emotions. In fact, our results represented that the insula and middle temporal gyrus displayed high centrality regions during discriminating emotions, and the two nodes showed distinguishing connection strength between the low‐ and high‐arousal of emotions. Therefore, the left insula and right middle temporal gyrus would serve as a crucial link connecting various nodes within the networks in the procedure of emotional discrimination.

Additionally, evidence based on the bipolar arousal‐valence model for emotion processing has shown that arousal is not separable from valence in its ability to predict arousal‐related neural activity.^[^ ^13^ ^]^ Consequently, it is challenging to investigate the impact of arousal alone without valence, although a meta‐regression analysis revealed that the arousal values were similar in the positive‐ and negative valences and unrelated to the paradigm tasks. The same is true for the valence value.^[^ ^37^ ^]^ In our paradigm, whether in the low‐arousal group or the high‐arousal group, we tried to compare the sequenced facial emotions with positive and negative valences in a balanced order, which would eliminate the impact of valence as much as possible. Nevertheless, the influence of emotional valence could not be completely excluded from the neural mechanism underlying the differences between the low‐arousal and high‐arousal states. In this work, we do not assert that arousal cannot exist independently of valence. Instead, our argument focuses on the interpretation of arousal within the framework of affective discrimination. Alternatively, valence may be one of the essences underlying discrimination of arousal‐dependent emotions.

Among these brain regions responsible for discriminating facial emotions, our data also showed that five regions, including the left inferior frontal gyrus, right angular gyrus, left and right supramarginal gyrus, and left fusiform gyrus, were specifically responsible for discriminating emotions regardless of arousal levels, as long as the faces were different (Figure S3, Supporting Information). ERPs of these subregions showed significant differences between the condition SESE and DFDE, whereas showed no change between the condition SFSE and SFDE. In contrast, the five sites seemed to be involved in processing the facial incongruencies rather than arousal‐dependent emotional incongruencies, with attention in dual‐feature tasks. Especially, the left inferior frontal gyrus and right angular gyrus emerged earlier than the other three regions after S2 onset, that is, for low‐arousal emotions, the right angular gyrus initiated at 223 ms, followed by the left inferior frontal gyrus initiated at 237 ms; and for high‐arousal emotions, the left inferior frontal gyrus began at 218 ms after the S2 onset, followed by the right angular gyrus at 230 ms. Iarrobino et al. utilized the transcranial direct current stimulation proved that the cathodal (inhibitory) stimulation applied to the right inferior frontal operculum affected the recognition of a high arousal emotion (i.e., anger), while cathodal stimulation applied to the left inferior frontal operculum modulated recognition of a low arousal emotion.^[^ ^11^ ^]^ The right inferior frontal gyrus participating in discriminating high‐arousal emotions was consistent with our finding (after 300 ms of S2 onset, Figure 2D), while our data showed the left inferior frontal was also involved in the high arousal group. However, their tasks only contained different faces and could have a conclusion with mixed effects of the facial and emotional incongruencies. Therefore, our findings indicate that the five brain regions mentioned above might play a critical role in the discrimination of both facial and emotional features.

Notably, SEEG recordings in patients with epilepsy allowed this extraordinary opportunity to study the neural mechanism of discrimination of arousal‐dependent emotions in the human brain. However, some limitations must be acknowledged. First, the spatial coverage of SEEG electrodes in patients is uneven and limited across the neocortex, as the surgical targets were tailored to meet specific clinical needs for each epilepsy cohort. The most critical limitation of SEEG is: an integrated cognition activity needed to map a large number of recording contacts, the need to cover wide parts of the brain, even if SEEG represents a very promising technique due to its relatively low invasiveness and high resolution of time and space. Consequently, it does not exclude the possibility that the current study is not unique to discriminating arousal‐dependent emotions, suggesting a potential universality for emotional processing. Second, we enrolled patients with electrodes without abnormal EEG signals during the entire recording period as the normal group, because it is impossible to obtain SEEG data from healthy people. To avoid the problem of facial or emotional recognition in some patients, all participants in this study passed the five‐emotion test with high accuracy (> 90%). On the other hand, Rigorous criteria of site screening and measurements were taken to ensure that the study reflected conditions as close to a normal human brain as possible. Third, while the SEEG data provided insights into the 11‐node network involved in cognitive activity, the findings would be further validated by applying intracranial modulation to target these nodes. Therefore, future studies should explore this approach. Nonetheless, our results investigated the mechanism underlying the discrimination of arousal‐dependent emotions using electrophysiological approaches with precise locations and high temporal resolution.

## 4 Conclusion

In summary, this study provides the pioneering demonstration of the basic distribution and connection modes of brain regions participating in the discrimination of low‐ and high‐arousal emotions. Our findings highlight distinct neural pathways for processing incongruent emotions based on arousal levels. This can enhance our understanding of how the brain discriminates between low‐ and high‐arousal emotions, which is crucial for developing targeted interventions for emotional disorders.

## 5 Experimental Section

### Participants

Patients with pharmacologically resistant epilepsy underwent surgical implantations of intracranial SEEG electrodes for diagnostic purposes as a part of their evaluation for epilepsy treatment in the Epilepsy Center of Xuanwu Hospital. Patients with abnormal brain structures or destructive lesions were excluded from this study. Patients who had clinical seizures that occurred within twenty‐four hours before the experimental tasks or during the experiment were also excluded. In total, 23 patients were recruited. After the task performance, four patients were excluded due to their low task accuracy (<60%). Therefore, SEEG recordings obtained from 19 patients (13 men; mean age = 29.26 years; Table S2, Supporting Information) were included for analysis. The study was approved by the Ethics Committee of the Xuanwu Hospital, Capital Medical University (Approval number: [2021]086); further, each patient provided informed consent to participate in the research.

### Experimental Tasks

Delayed‐matched/mismatched sample paradigms were used. In each trial, a pair of pictures were sequentially presented on a gray background on a standard liquid crystal dispaly screen, with each picture adjusted to an average visual angle of 2.1 degrees at a viewing of ≈50 cm. Presented pictures included black‐and‐white female faces with emotions including happiness, sadness, anger, fear, and a neutral face. Standard facial expression images were obtained from the Japanese Female Facial Expression dataset. The images containing semantic rating data are from psychological experiments (Table S3, Supporting Information). Images were chosen according to their predominant emotions (with the highest rating in six scales) in low‐ and high‐arousal with distinct semantic ratings from each other, respectively.

The task was divided into two runs, each consisting of 216 trials. One run involved low‐arousal emotions, including happiness and sadness, mixed with neutral emotions. The other run involved high‐arousal emotions, including anger and fear, mixed with neutral emotions. Each pair of pictures consisted S1 and S2, which were presented for 300 ms each with an interstimulus interval of 500 ms. The interval between each pair of stimuli was 3.6 s. The sequence and frequency of each emotional face in each run were counterbalanced. To minimize the potential impact of valence on the cognitive processes during discriminating arousal‐dependent emotions, the sequence of appearance was balanced for positive‐ and negative‐valence emotions, that is, the influence of S2 presenting a positive emotion on S1 presenting a negative emotion is offset by the influence of S2 presenting a negative emotion on S1 presenting a positive emotion through this balancing process.

Participants were encouraged to maintain focus on the center of the screen, to judge whether the emotion of S2 was identical to the emotion of S1, and to indicate their answer of “yes” or “no” by pressing “F” or “J” on the keyboard, respectively. Additionally, the task included four conditions, that is the condition SFSE, SFDE, DFSE, and DFDE. The stimuli were presented using E‐Prime software (version 2.0; Psychology Software Tools, Inc., Sharpsburg, PA, USA). Participants were instructed to respond as quickly and accurately as possible. The correct key button presses (left and right) were counter‐balanced. All participants had normal or corrected‐to‐normal vision and were right‐handed.

### SEEG Recordings

A semirigid platinum/iridium SEEG electrode (Sinovation [Beijing] Medical Technology Co., Ltd., Beijing, China), with a diameter of 0.8 mm, contact length of 2 mm, contact interval of 1.5 mm, and contact number of 8–20, was implanted as needed. Electrode placement was based only on clinical requirements and was not affected by the study needs. A Blackrock Microsystem (Blackrock Microsystems LCC, Salt Lake City, UT, USA) was used for recording. During the SEEG recording, the EEG signals were grounded to a vertex/subdermal screw, referenced to the recording site within the low‐activity white matter, sampled at 2000 Hz, and filtered between 0.05 and 500 Hz. A notch filter was applied at a frequency of 50 Hz. The electrical pulse triggered by each S2 onset was recorded along with the SEEG signal for precise synchronization.

### Electrode Reconstruction and Site Definition

As previously described, SEEG electrodes were reconstructed using Brainstorm.^[^ ^38^ ^]^ For each patient, a T1‐weighted 1‐mm isometric structural magnetic resonance imaging (MRI) scan was obtained before electrode implantation, and a Siemens computed tomography (CT) scan was obtained after implantation. The post‐implantation CT was then reregistered with the pre‐implantation anatomical MRI scan using sequential pattern mining algorithms. This registration allowed visualization of the electrode contacts on top of the MRI scan. Electrode contacts in the native space were transformed into the Montreal Neurological Institute (MNI) space using the MNI152 structural template volume image. The obtained MNI coordinates indicated the localization of electrode contacts, and the subregions in the human brain were defined using the Human Brainnetome Atlas, which provides 246 fine‐grained cortical subregions.^[^ ^39^ ^]^ The BrainNet Viewer tool was used to visualize these sites within the human brain subregions.^[^ ^40^ ^]^

### Statistical Analysis

Rigorous screening criteria were applied for recording sites to approximate normal conditions as closely as possible. Based on a presurgical evaluation by neurological and neurosurgical experts, recording sites containing ictal and interictal activities were excluded. EEG signals with abnormal noise or artifacts were excluded. Sites located within the white matter or the cerebrospinal fluid were excluded. The eligible data were all from high‐accuracy (> 60%) performances during the cognitive tasks. A threshold for each ERPs trace was set three standard deviations from the mean baseline, which was calculated across all trials. ERPs signals out of the threshold would be excluded. All the data were visually rechecked on a trial‐by‐trial basis. Only sites with more than 30 trials per condition were included in further analysis. Finally, 19 patients were included, with 181 electrodes implanted, containing a total of 2148 recording contacts. The number of contacts located in the white matter was 876. Thirty‐nine sites with artifacts and 647 sites with epileptiform activities were also excluded. Finally, 137 electrodes containing 586 recording sites were selected for further analysis (for a detailed site screening, see Figure S4, Supporting Information). Table S1 (Supporting Information) presents the distribution of sites within the subregions.

Behavioral data were exported using E‐Prime software (version 2.0; Psychology Software Tools, Inc., Sharpsburg, PA, USA) and analyzed using GraphPad Prism (version 8.0; GraphPad Software). A paired *t*‐test was used to compare the differences in reaction times and accuracies between the low‐arousal and high‐arousal groups in each condition. The data were plotted as the mean ± standard error of the mean (SEM).

The SEEG data were preprocessed using Brainstorm. For the ERP analysis, the signals were resampled to 1000 Hz and digitally filtered with a bandpass filter of 1–200 Hz. The 50‐Hz power line interference (including its harmonics) was removed from the data. The epochs were extracted from 200 ms pre‐S1 to 1500 ms post‐S2, with a total duration of 2500 ms for each epoch trial. The baseline was corrected using the average from the 200 ms pre‐S1 to the 1 ms pre‐S1 interval. The permutation test was used to examine the differences in SEEG amplitudes between conditions.

To measure the connectivity between the subregions, the averaged ERP for each site in each condition (SFDE or DFDE) was subtracted from the corresponding ERP in the SFSE condition to highlight the activities associated with incongruency processing. Next, Spearman's correlation was calculated for each pair of sites using the subtracted averaged ERP. The correlation coefficient between the two sites was considered indicative of connectivity only when *p* < 0.05, as determined by the Spearman correlation analysis. Connectivity between the two subregions was represented by the mean correlation coefficient of the sites within the corresponding subregional groups. The Mann–Whitney *U* test was used to compare the connectivity coefficients within two individual subregions between low‐arousal and high‐arousal emotional stimuli. For the averaged coefficient of subregions, a mixed‐effects model (restrict maximum likelihood) was used to analyze the effect of “time” and “emotional arousal” on the integral connections within core subregions. To reduce the chances of obtaining false‐positive test results, a false discovery rate (FDR) correction was used to adjust for multiple comparisons. All statistical tests were two‐sided unless stated otherwise.

The global efficiency (*E_glob_*) and clustering coefficient were used to characterize the networks. The *E_glob_*, which described the averaged minimum links of the network, was calculated as follows:

(1) $$E_{glob}=\frac{1}{N\left(N-1\right)}\sum_{i,j\in V,i\neq j}\frac{1}{l_{ij}}$$

here, *l_ij_* represents the minimum number of links between node *i* and node *j*, and *N* is the total number of nodes within the network. If there is no connection between the two nodes, then $\frac{1}{l_{ij}}$ should be considered as zero.

The clustering coefficient, which represented the grouping of the network, was calculated as follows:

(2) $$CC=\frac{1}{N}\sum_{i\in V}C_{i}$$

here, CC represents the averaged clustering coefficient of each node within the network. The C_i_ is the ratio of the actual links between the node *i* and its neighborhood nodes *j* and node *m*, divided by the number of links that could potentially exist between them.

## Conflict of Interest

The authors declare no conflict of interest.

## Supporting information



Supporting Information

## Data Availability

The data that support the findings of this study are available from the corresponding author upon reasonable request.
