# Supplementary material for: Left Insula and Right Middle Temporal Gyrus Dominate Cortical Network Discriminating Arousal‐dependent Emotions
Source: Adv Sci (Weinh). 2025 Jan 17;12(10):2411790. doi: 10.1002/advs.202411790 (PMC11905088; doi:10.1002/advs.202411790)
Supplement: Supplementary file 1 — Supporting Information [file ADVS-12-2411790-s001.docx]

Fig S1. ERPs of subregions specifically participating in processing low-arousal and high-arousal emotion incongruencies.

**A-C**, representative ERPs of subregions that participating in processing emotion incongruencies under the same face, as condition SFDE v.s. SFSE. ERPs of Subregions that participating in both low-arousal and high-arousal emotional incongruency processing were shown in (**A**); ERPs of subregions that participating only in low-arousal emotional incongruency processing were shown in (**B**); and ERPs of subregions that participating only in high-arousal emotional incongruency processing were shown in (**C**).

**D-F,** representative ERPs of subregions that participating in processing emotion incongruencies under different faces, as condition DFDE v.s. SFSE. ERPs of Subregions that participating in both low-arousal and high-arousal emotional incongruency processing were shown in (**D**); ERPs of subregions that participating only in low-arousal emotional incongruency processing were shown in (**E**); ERPs of subregions that participating only in high-arousal emotional incongruency processing were shown in (**F**).

The averaged ERPs traces of trials were indicated as solid line and SEM was shown as the shadow. The gray vertical rectangles indicated the durations of S1 and S2, respectively. FDR corrected multiple t-test was applied between condition SFSE (light color), and SFDE (dark color in **A-C**), or DFDE (dark color in **D-F**) after S2 onset, and black horizontal bars indicated corrected *P* < 0.05 lasting for at least 20 ms.

Abbreviation: AG, angular gyrus; AI, anterior insula; AMY, amygdala; FG, fusiform gyrus; IFG, inferior frontal gyrus; HIP, hippocampus; MFG, middle frontal gyrus; MTG, middle temporal gyrus; PCC, posterior cingulate cortex; PI, posterior insula; preCG, precentral gyrus; SFG, superior frontal gyrus; SG, supramarginal gyrus; SPL, superior parietal lobule; supOG, superior occipital gyrus.


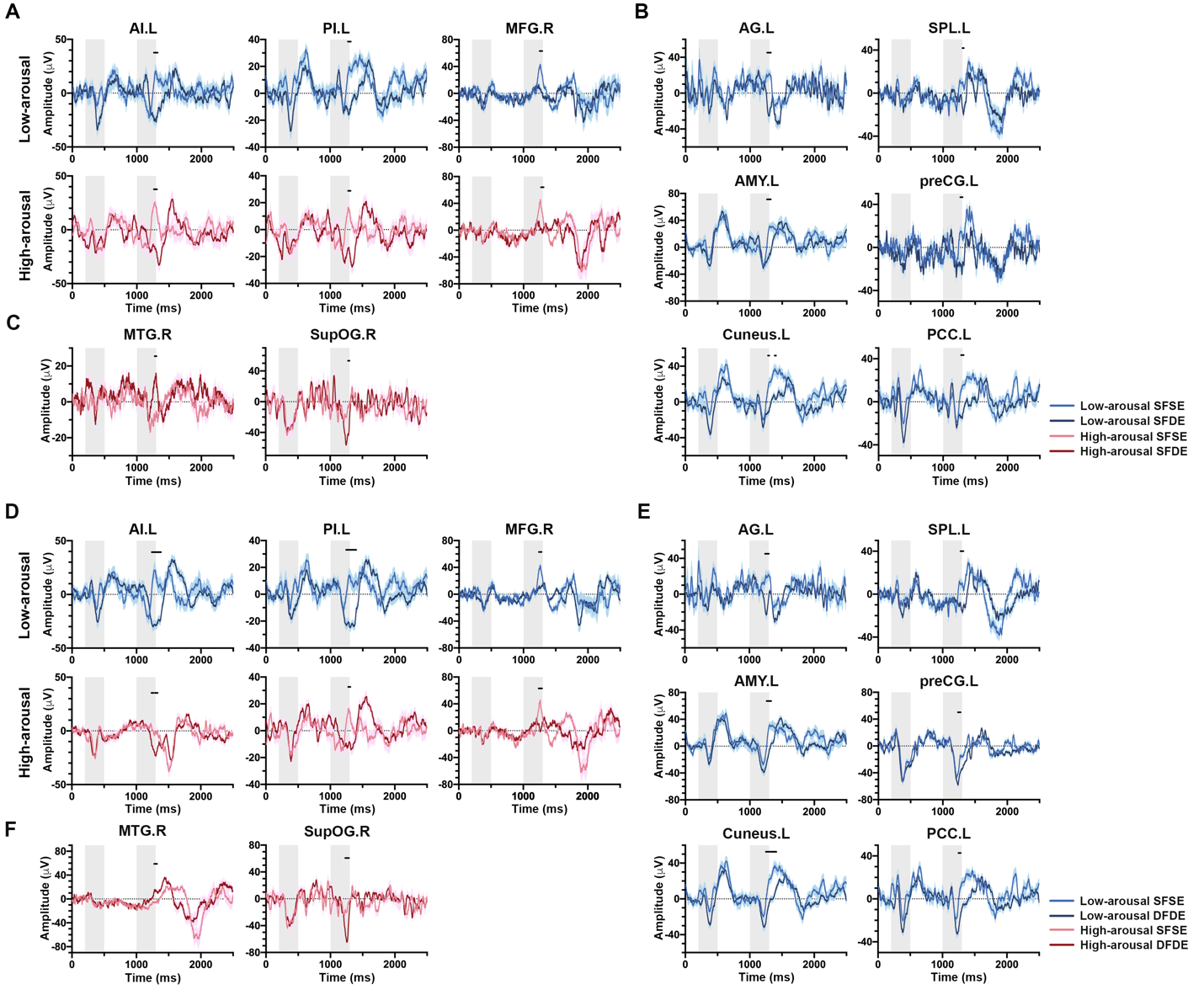


Fig S2. Histograms of correlation coefficient between subregions in low- and high-arousal emotional incongruency processing.

**A-C**， correlation coefficients between sites locating in each pair of subregions， which participating in low-arousal (blue) and high-arousal (red) emotional incongruency processing in condition SFDE, during 101-200 ms (**A**), 201-300 ms (**B**), and 301-400 ms (**C**) from S2 onset.

**D-F**, correlation coefficients between sites locating in each pair of subregions， which participating in low-arousal (blue) and high-arousal (red) emotional incongruency processing in condition DFDE, during 101-200 ms (**D**), 201-300 ms (**E**), and 301-400 ms (**F**) from S2 onset.

Data were presented as mean ±SEM after a Mann-Whitney test. **P* < 0.05, ***P* < 0.01, and ****P* < 0.001.


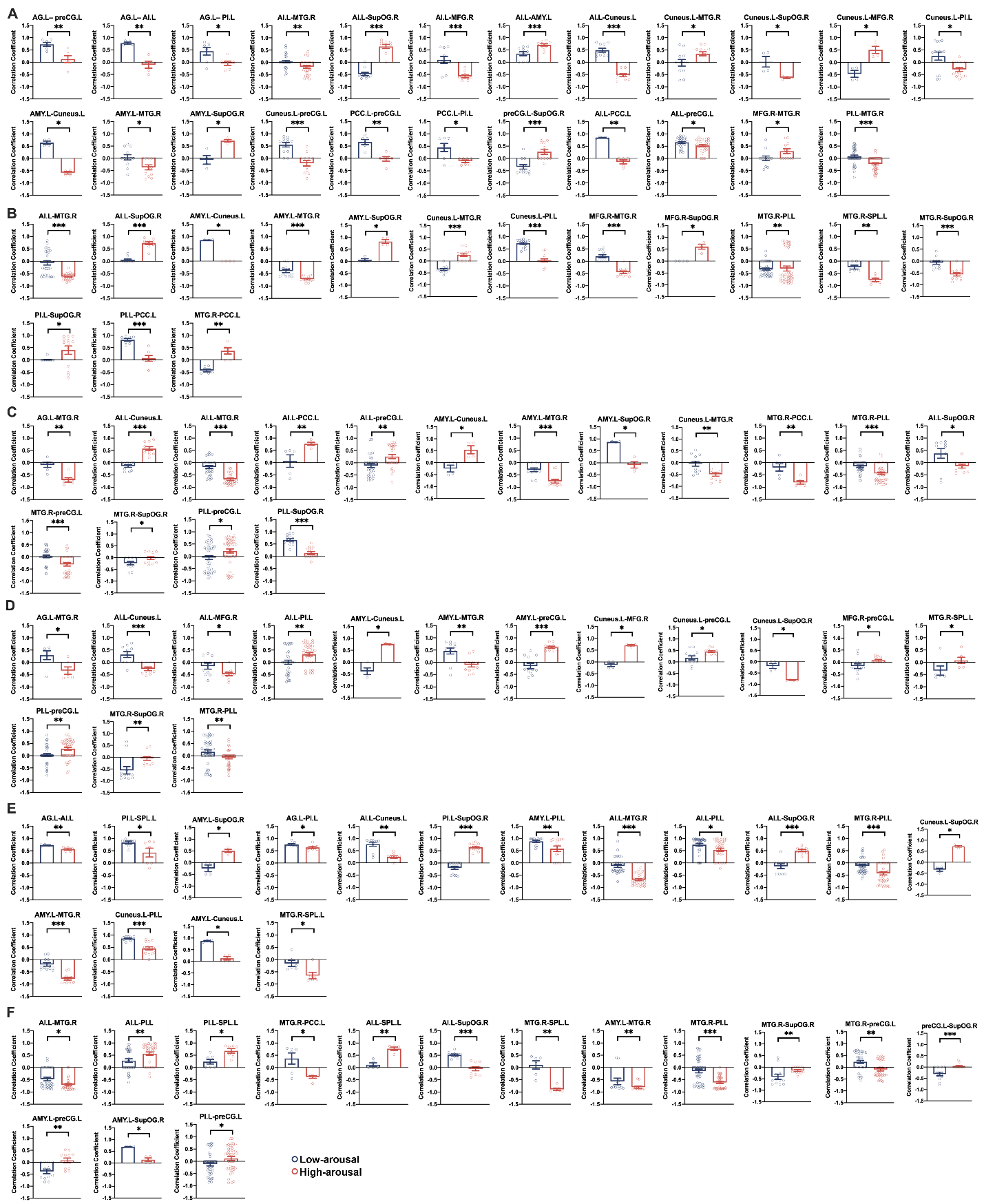


Fig S3. ERPs of subregions chironologicaly participating in discriminating arousal-independent emotions under different faces.

**A**, spatial distributions of five key nodes (green ball), and the sequency of discriminating low-arousal (upper panel) and high-arousal (bottom panel) emotions under conditions of different human faces, respectively. Black arrows represent the sequence of processing.

**B**, representative ERPs of five nodes that participating in processing low-arousal (blue) and high-arousal (red) emotion incongruencies under different faces, as condition DFDE v.s. SFSE. The averaged ERPs traces of trials were indicated as solid line and SEM was shown as the shadow. The gray vertical rectangles indicated the durations of S1 and S2, respectively. FDR corrected multiple t-test was applied between condition SFSE (light color) and DFDE (dark color) after S2 onset, and black horizontal bars indicated corrected P < 0.05 lasting for at least 20 ms.

Abbreviation: AG, angular gyrus; FG, fusiform gyrus; IFG, inferior frontal gyrus; SG, supramarginal gyrus.


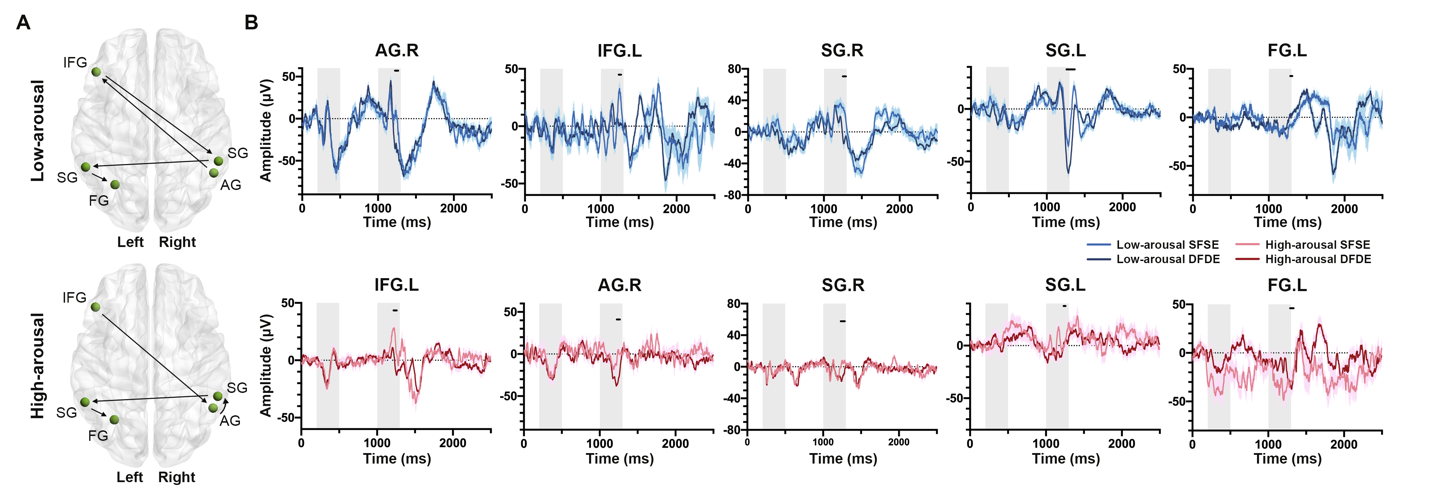


Fig S4. Experimental flow chart.


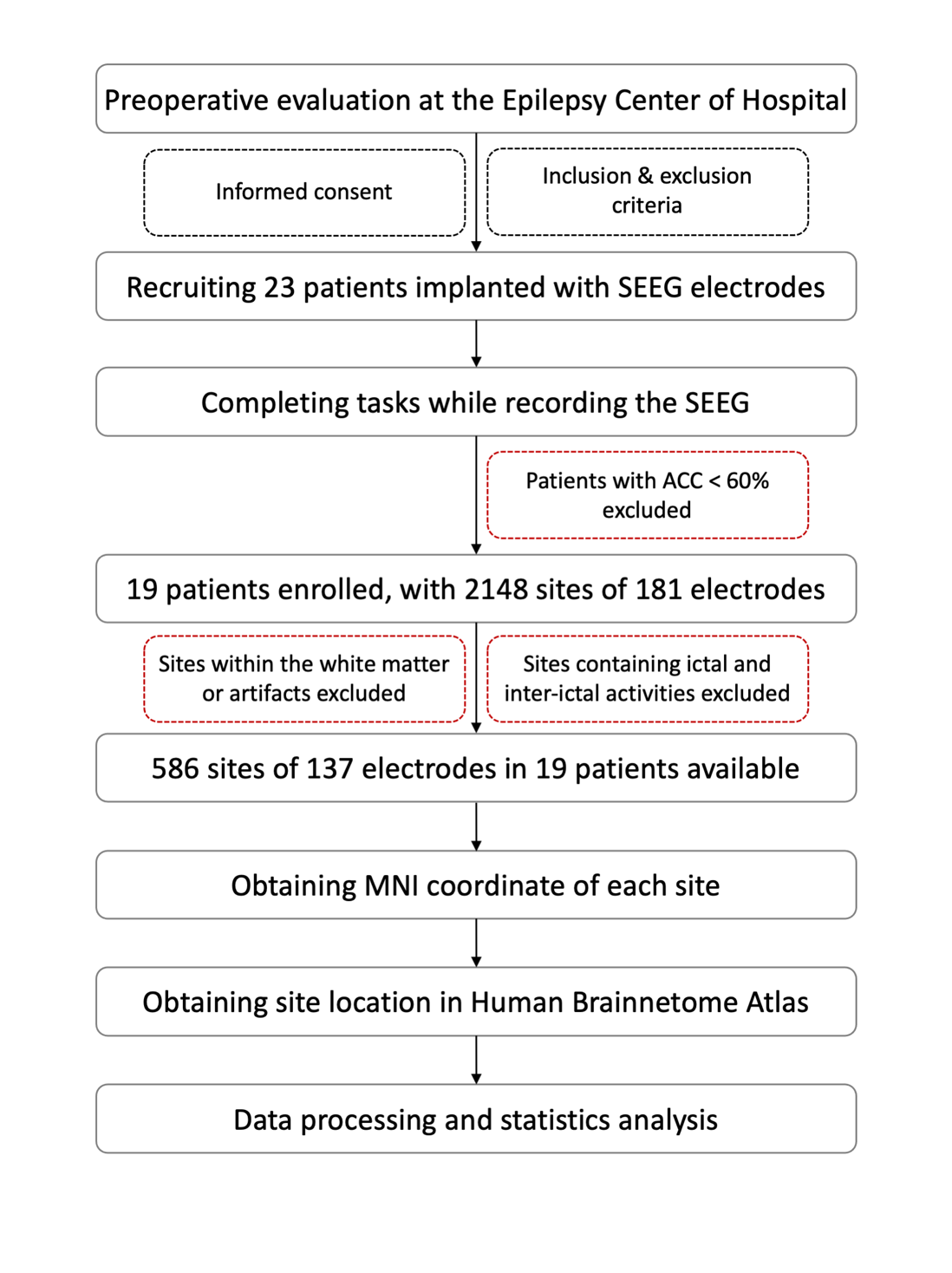


Table S1. Subregions of individual cortical lobes locating the recording sites.

| Lobes | Subregions | Site counts  (Left hemisphere) | Site counts  (Right hemisphere) |
| --- | --- | --- | --- |
| Frontal lobe |  | **101** | **104** |
|  | Middle Frontal Gyrus  Inferior Frontal Gyrus  Precentral Gyrus  Orbital Gyrus  Superior Frontal Gyrus | 59  13  19  9  1 | 56  19  14  7  8 |
| Parietal lobe |  | **32** | **44** |
|  | Postcentral Gyrus  Supramarginal Gyrus  Angular Gyrus  Precuneus  Superior Parietal Lobule | 11  13  4  1  3 | 5  26  12  1  0 |
| Temporal Lobe |  | **95** | **90** |
|  | Superior Temporal Gyrus  Middle Temporal Gyrus  Inferior Temporal Gyrus  Parahippocampal Gyrus  Posterior Superior Temporal Sulcus  Fusiform Gyrus | 13  49  12  1  5  15 | 4  58  16  4  1  7 |
| Insular lobe |  | **31** | **22** |
|  | Posterior insula  Anterior insula | 9  22 | 8  14 |
| Limbic lobe |  | **17** | **2** |
|  | Anterior Cingulate Gyrus  Middle Cingulate Gyrus  Posterior Cingulate Gyrus | 8  4  5 | 2  0  0 |
| Occipital lobe |  | **15** | **3** |
|  | Occipital Gyrus  Cuneus  Superior Occipital Gyrus | 9  6  0 | 1  0  2 |
| Subcortex |  | **15** | **15** |
|  | Amygdala  Hippocampus | 2  13 | 2  13 |

Table S2. SEEG recording information of 19 participants.

| Subject NO. | Sex (F/M) | Age (years) | Available sites count | Available electrodes count |
| --- | --- | --- | --- | --- |
| 1# | M | 18 | 38 | 10 |
| 2# | F | 27 | 59 | 10 |
| 3# | M | 22 | 21 | 8 |
| 4# | M | 35 | 14 | 6 |
| 5# | M | 18 | 10 | 4 |
| 6# | M | 30 | 1 | 1 |
| 7# | F | 36 | 27 | 5 |
| 8# | M | 25 | 29 | 7 |
| 9# | M | 23 | 51 | 9 |
| 10# | M | 26 | 18 | 6 |
| 11# | F | 38 | 21 | 9 |
| 12# | F | 23 | 37 | 8 |
| 13# | M | 37 | 36 | 8 |
| 14# | M | 33 | 78 | 12 |
| 15# | F | 34 | 23 | 5 |
| 16# | F | 31 | 10 | 5 |
| 17# | M | 22 | 30 | 7 |
| 18# | M | 30 | 29 | 6 |
| 19# | M | 48 | 54 | 11 |

Table S3: Mean sematic rating of images in the paradigm.

| Face NO. | Emotion | Mean sematic rating | | | | | |
| --- | --- | --- | --- | --- | --- | --- | --- |
|  |  | **Happy** | **Sad** | **Angry** | **Fear** | **Surprise** | **Disgusting** |
| 1 | **happy** | 4.77 | 1.29 | 1.26 | 1.23 | 2.45 | 1.23 |
| 1 | **sad** | 1.26 | 4.13 | 2.4 | 2.74 | 1.77 | 3.1 |
| 1 | **angry** | 1.55 | 1.9 | 4.32 | 1.81 | 2.1 | 3.9 |
| 1 | **fear** | 1.32 | 4.16 | 1.97 | 4.26 | 3.65 | 2.87 |
| 1 | **neutral** | 2.84 | 1.94 | 1.77 | 1.87 | 2.13 | 1.68 |
| 2 | **happy** | 4.42 | 1.55 | 1.42 | 1.45 | 1.42 | 1.45 |
| 2 | **sad** | 1.39 | 4.42 | 2.77 | 3.81 | 1.94 | 3.68 |
| 2 | **angry** | 1.68 | 3.32 | 4.1 | 2.71 | 2.1 | 3.9 |
| 2 | **fear** | 1.26 | 3.03 | 2.94 | 4.1 | 4.19 | 4.45 |
| 2 | **neutral** | 1.84 | 2.29 | 2.35 | 2.13 | 3.71 | 2.32 |
| 3 | **happy** | 4.9 | 1.39 | 1.32 | 1.35 | 1.29 | 1.29 |
| 3 | **sad** | 1.55 | 4.32 | 1.71 | 2.55 | 1.35 | 2.74 |
| 3 | **angry** | 1.19 | 2.16 | 4.74 | 1.39 | 1.55 | 2.84 |
| 3 | **fear** | 1.29 | 3.13 | 2.55 | 4.19 | 4.52 | 3.45 |
| 3 | **neutral** | 3.03 | 2.45 | 2 | 1.77 | 1.74 | 1.9 |

Note: The images containing sematic rating data are from psychological experiments. The expression labels on the images just represent the predominant expression in that image - the expression that the subject was asked to pose.
